# Hybridization and adaptive introgression in a marine invasive species in native habitats

**DOI:** 10.1016/j.isci.2023.108430

**Published:** 2023-11-10

**Authors:** José Martin Pujolar, Denise Breitburg, Joanna Lee, Mary Beth Decker, Cornelia Jaspers

**Affiliations:** 1 Centre for Gelatinous Plankton Ecology and Evolution, DTU Aqua - Technical University of Denmark, 2800 Kgs. Lyngby, Denmark; 2 Smithsonian Environmental Research Center, Edgewater, MD 21037, USA; 3 Boston University, Biology, Boston, MA 02215, USA; 4 Department of Ecology and Evolutionary Biology, Yale University, New Haven, CT 06520-8106, USA

**Keywords:** Environmental science, Nature conservation, Evolutionary biology

## Abstract

Hybridization of distinct populations or species is an important evolutionary driving force. For invasive species, hybridization can enhance their competitive advantage as a source of adaptive novelty by introgression of selectively favored alleles. Using single-nucleotide polymorphism (SNP) microarrays we assess genetic diversity and population structure in the invasive ctenophore *Mnemiopsis leidyi* in native habitats. Hybrids are present at the distribution border of two lineages, especially in highly fluctuating environments including very low salinities, while hybrids occur at lower frequency in stable high-saline habitats. Analyses of hybridization status suggest that hybrids thriving in variable environments are selected for, while they are selected against in stable habitats. Translocation of hybrids might accelerate invasion success in non-native habitats. This could be especially relevant for *M. leidyi* as low salinity limits its invasion range in western Eurasia. Although hybridization status is currently disregarded, it could determine high-risk areas where ballast water exchange should be prevented.

## Highlights

• SNP-chip detects *M. leidyi* hybrids in native habitats including low saline areas
• Hybrid analysis suggests selection against admixed individuals in stable habitats
• Hybrid prevalence in fluctuating, low saline areas suggests a selective advantage
• Hybrid translocation from low saline areas could facilitate successful invasions

## Graphical abstract

**Figure:**
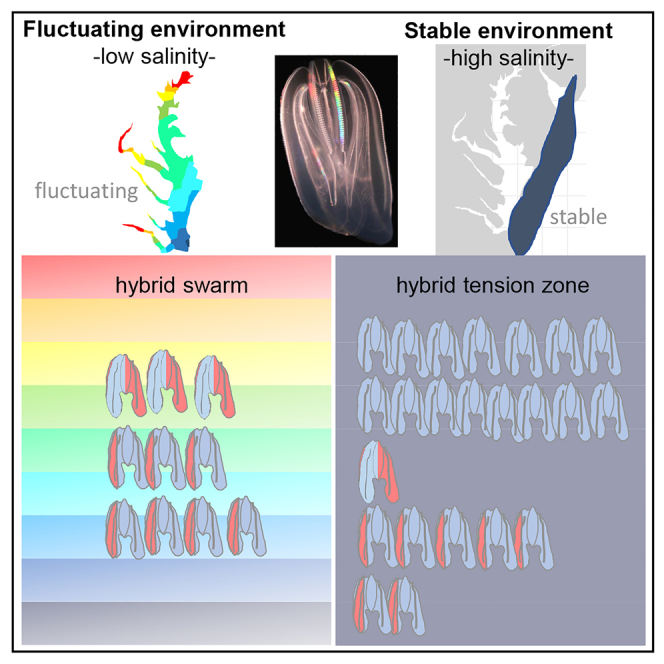

## Introduction

Marine invasive species cause large biological and economic impacts worldwide.^1^^,^^2^ Records of new non-indigenous species sightings are increasing without signs of saturation either now^3^^,^^4^ or in projections.^5^ It is still debated which traits allow species to invade ecosystems and which factors facilitate invasion success.^6^ It is acknowledged, however, that hybridization with successful interbreeding of distinct populations or species in the recipient ecosystem can accelerate invasion success.^7^ During hybridization, admixed genotypes are formed^8^ and adaptive novelty can be attained due to the introgression of selectively favored alleles.^9^^,^^10^^,^^11^ Invasion success has been shown to be higher for hybrid plant populations formed in the recipient habitat,^7^ but the effect of hybridization within native populations, setting the stage for increased fitness and invasion potential, remains understudied, especially for marine species.

In native habitats, admixed populations can be found in hybrid zones, i.e., the area where two lineages meet naturally. The geographic extent of hybrid zones is normally narrow and maintained by balancing dispersal, selection, hybrid fitness, and ecological conditions.^9^^,^^12^^,^^13^ Analysis of population genomic data from hybrid zones offer the opportunity to study the evolutionary consequences of introgression.^14^^,^^15^^,^^16^^,^^17^ Hybrids can show either increased (hybrid vigor) or decreased (outbreeding depression) fitness.^8^^,^^18^ While in many cases hybrids are outperformed by pure lines, hybrid genotypes have been shown to perform better under novel environmental conditions or in extreme habitats,^19^ as exemplified for alpine-adapted butterflies^20^ and North Atlantic eels in Iceland.^21^

**Figure 1:**
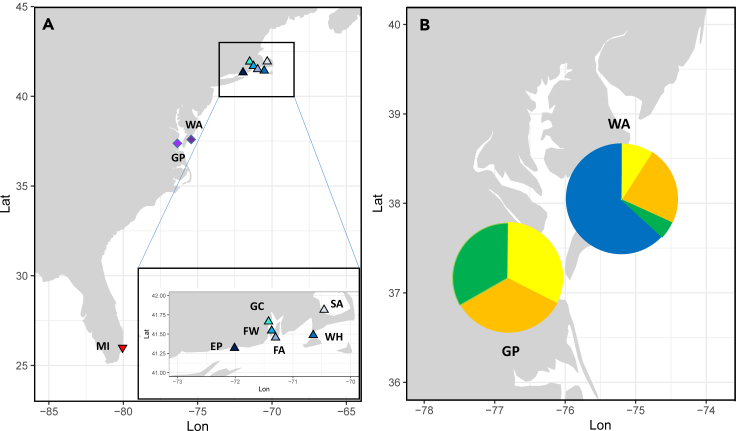
*Mnemiopsis leidyi* sampling locations, USA east coast (2018/2020), and hybrid population classification (A) Northern populations (blue triangle up) with stations (WH = Woods Hole, MA; SA = Sandwich, MA; EP = Long Island Sound, Esker Point, CT and Narraganset Bay, RI with FA = Fort Adams, FW = Fort Wetherill, GC = Greenwich Cove), hybrid populations (pink diamond) with Chesapeake Bay (GP = Gloucester Point, VA) and Atlantic coast (WA = Wachapreague, VA). Southern population (red triangle down) with MI = Miami, FL; Cape Hatteras indicates population border (hatched line). (B) Hybrid populations with proportion of pure northern (blue), F_2_/F_x_-hybrids (green), 1^st^ generation (yellow) and 2^nd^ generation (orange) backcrosses inside and outside of Chesapeake Bay, 2018.

The ctenophore *Mnemiopsis leidyi* provides a case to test the role of hybridization and introgression as a potential source of adaptive novelty in the context of biological invasion. The species is native to the Atlantic coasts of North and South America.^22^ It was introduced from the east coast of the United States to western Eurasia, and is now invasive in large areas of the Black Sea, the Caspian Sea, and the Mediterranean, as well as NW Europe.^23^ Previous genetic studies identified two distinct populations, or lineages, in the native range: a southern lineage occurring in Florida/Gulf of Mexico and a northern lineage occurring in New England.^24^^,^^25^ Based on mitochondrial cytochrome *b* and six nuclear microsatellite loci, Bayha et al.^25^ identified Cape Hatteras as the location of a genetic break between the two lineages driven by oceanographic features.^26^ Using whole genome data, Jaspers et al.^27^ reported high genetic differentiation between the two lineages (Figure 1), which is comparable to differentiation between named congeneric species.^28^

Here, we use a diagnostic single-nucleotide polymorphism (SNP) microarray developed for the northern and southern *M. leidyi* lineages^29^ to study spatial and temporal genetic diversity, as well as population structure, of *M. leidyi* in its native habitat along the US Atlantic coast. Estuaries in the northwestern Atlantic experience differing salinity conditions, especially Chesapeake Bay, the largest estuary in the US, which has a large spatial and temporal variation in environmental conditions including extremely low salinity levels.^30^ We investigate the level of hybridization and introgression in eight locations and link occurrence of hybrids to environmental conditions and oceanographic features/dispersal as well as potential evolutionary processes.

## Results

### Population structure

SNP genotyping of 176 *M. leidyi* individuals from eight locations in two regions (New England and mid-Atlantic Coast) was conducted (Figure 1). For comparison, one location from Miami, FL was also included. Measures of genetic variability are summarized in Table S1. Observed heterozygosities ranged from 0.27 to 0.36 with no statistical differences observed among samples (p = 0.921). Similarly, no differences were found in expected heterozygosities (p = 0.948). Allelic richness ranged from 1.74 in Greenwich Cove to 1.91 in Sandwich, with no differences observed among samples (p = 0.696).

No temporal genetic differentiation was detected at the four locations, where samples were collected in both 2018 and 2020: Sandwich (F_ST_ = 0.017; p = 0.054), Woods Hole, (F_ST_ = −0.015; p = 0.703), Fort Adams (F_ST_ = 0.005; p = 0.198), and Fort Wetherill (F_ST_ = −0.027; p = 0.856). Therefore, temporal samples within locations were pooled for further analyses.

**Figure 2:**
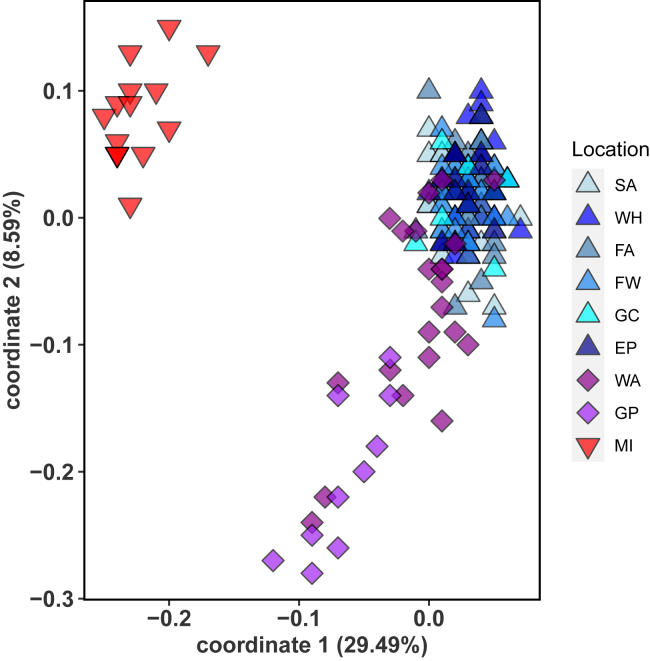
Principal component analysis (PCA) for *Mnemiopsis leidyi* populations along the USA east coast (2018/2020) Northern populations (blue triangles): WH = Woods Hole, MA; SA = Sandwich, MA; EP = Long Island Sound, Esker Point, CT and Narraganset Bay, RI with FA = Fort Adams, FW = Fort Wetherill, GC = Greenwich Cove; hybrid populations (pink diamonds): Chesapeake Bay (GP = Gloucester Point, VA) and Atlantic coast (WA = Wachapreague, VA); and southern population (red downward triangles): MI = Miami, Florida).

A highly significant genetic differentiation was detected between the two Virginia locations (Wachapreague on the Altantic coast and Gloucester Point inside Chesapeake Bay) and all other samples, with no genetic structure detected for samples collected North of Virginia along the cost and estuaries of New England (Table 1). In accordance with the pairwise F_ST_ values, principal components analysis (PCA) indicated three clusters (Figure 2). One group included individuals from all northern sites, Sandwich, MA, Woods Hole, MA, and Esker Point, CT, as well as the Narraganset locations of Fort Adams, Fort Wetherill, and Greenwich Cove, RI. A second group included all individuals from Chesapeake Bay (Gloucester Point) and east of Chesapeake Bay along the Atlantic coast (Wachapreague). A third distinct cluster included all individuals from Miami, FL. The first two coordinates explained 29.5% and 8.6% of the variance, respectively (p < 0.001), while the other axes were not significant (p > 0.05), following Tracy-Widom statistics.

**Table 1:** Pairwise genetic differentiation (F_ST_) between sampling locations (lower diagonal) and p values (upper diagonal)

**F_ST_**	**Sandwich, MA**	**Woods Hole, MA**	**Fort Adams, RI**	**Fort Wetherill, RI**	**Greenwich Cove, RI**	**Esker Point, CT**	**Wacha-preague, VA**	**Gloucester Point, VA**	**Miami, FL**
**Sandwich**	∗∗∗∗∗	0.324	0.856	0.692	0.432	0.802	**<0.001**	**<0.001**	**<0.001**
**Woods Hole**	0	∗∗∗∗∗	0.748	0.739	0.162	0.964	**<0.001**	**<0.001**	**<0.001**
**Fort Adams**	−0.004	−0.003	∗∗∗∗∗	0.919	0.423	0.658	**<0.001**	**<0.001**	**<0.001**
**Fort Wetherill**	−0.002	−0.003	−0.005	∗∗∗∗∗	0.405	0.378	**<0.001**	**<0.001**	**<0.001**
**Greenwich Cove**	0	−0.007	−0.002	0	∗∗∗∗∗	0.533	**0.009**	**<0.001**	**<0.001**
**Esker Point**	−0.004	0.007	−0.002	0.002	0.002	∗∗∗∗∗	**<0.001**	**<0.001**	**<0.001**
**Wachapreague**	**0.03**	**0.031**	**0.026**	**0.024**	**0.028**	**0.022**	∗∗∗∗∗	**<0.001**	**<0.001**
**Gloucester P.**	**0.158**	**0.147**	**0.144**	**0.141**	**0.13**	**0.144**	**0.056**	∗∗∗∗∗	**<0.001**
**Miami**	**0.4**	**0.403**	**0.387**	**0.385**	**0.461**	**0.426**	**0.325**	**0.322**	∗∗∗∗∗

Significant values in bold.

**Figure 3:**
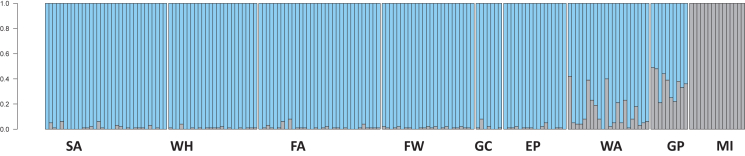
Admixture analysis visualized by STRUCTURE plots of *Mnemiopsis leidyi* sampled along the US east coast Individuals were assigned on the basis of the most likely K, in this case (K = 2). Locations as outlined in Figure 1 (SA = Sandwich, MA; WH = Woods Hole, MA; FA = Fort Adams, RI; FW = Fort Wetherill, RI; GC = Greenwich Cove, RI; EP = Esker Point, CT; WA = Wachapreague, VA; GP = Gloucester Point, Chesapeake Bay, VA; MI = Miami, FL).

**Figure 4:**
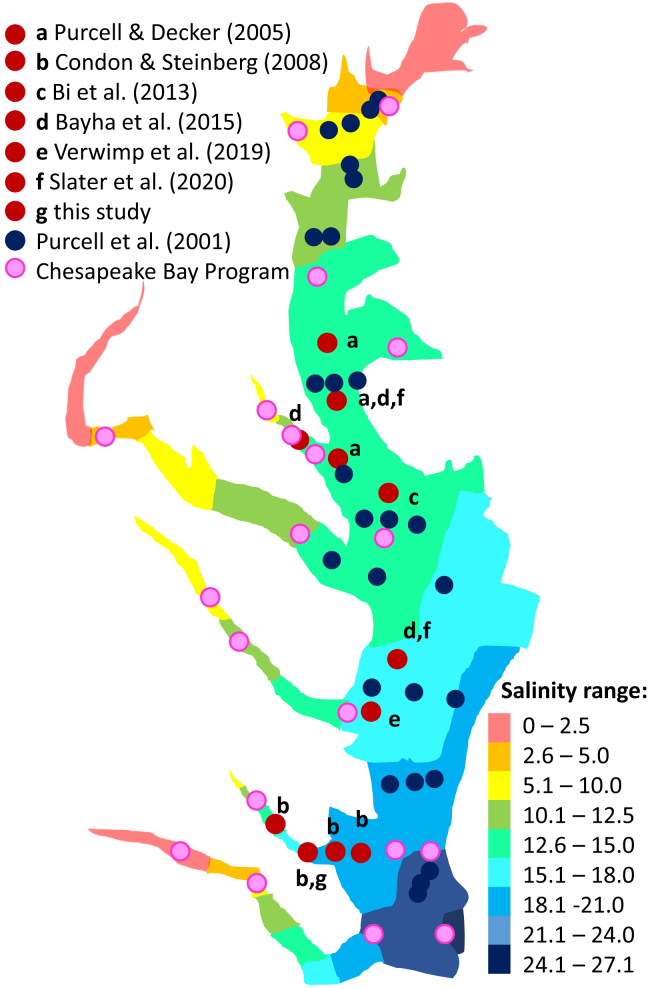
Average yearly surface salinity of Chesapeake Bay (1985–2018) with selected *M. leidyi* observations from literature Red dots from (a) Purcell and Decker (2005),^37^ (b) Condon and Steinberg (2008),^38^ (c) Bi et al. (2013),^39^ (d) Bayha et al. (2015),^25^ (e) Verwimp et al. (2019),^40^ (f) Slater et al. (2020),^41^ (g) this study; blue dots from Purcell et al. (2001)^42^; pink dots from Chesapeake Bay program—sourced 20.5.2023. Schematic map sourced and modified from Chesapeake Bay Program along with presence of *M. leidyi* data (www.chesapeakebay.net), sampling locations approximated.

STRUCTURE analysis suggested a scenario with two groups (K = 2) as the most likely (Figure 3), which correspond to the northern and southern lineages. We conducted structure analyses including K = 3 to 5, which did not result in any logical additional clustering (Figure S1). All individuals north of Chesapeake Bay could be assigned with high confidence to the northern lineage, while the baseline individuals from Miami could be assigned with high confidence to the southern lineage. All individuals north of Chesapeake Bay were suggested to be non-admixed, while the majority of individuals from the Chesapeake Bay area (coastal and Bay) were of admixed origin. At the Atlantic coast of Virginia (Wachapreague), 36.3% of individuals showed an admixture proportion >10%, while at the sample station inside Chesapeake Bay (Gloucester Point), 100% of individuals were admixed, with admixture proportion ranging from 21 to 49%. Inside Chesapeake Bay, *M. leidyi* have also been confirmed from large salinity ranges, including very low salinities (Figure 4).

### Hybrid identification

**Figure 5:**
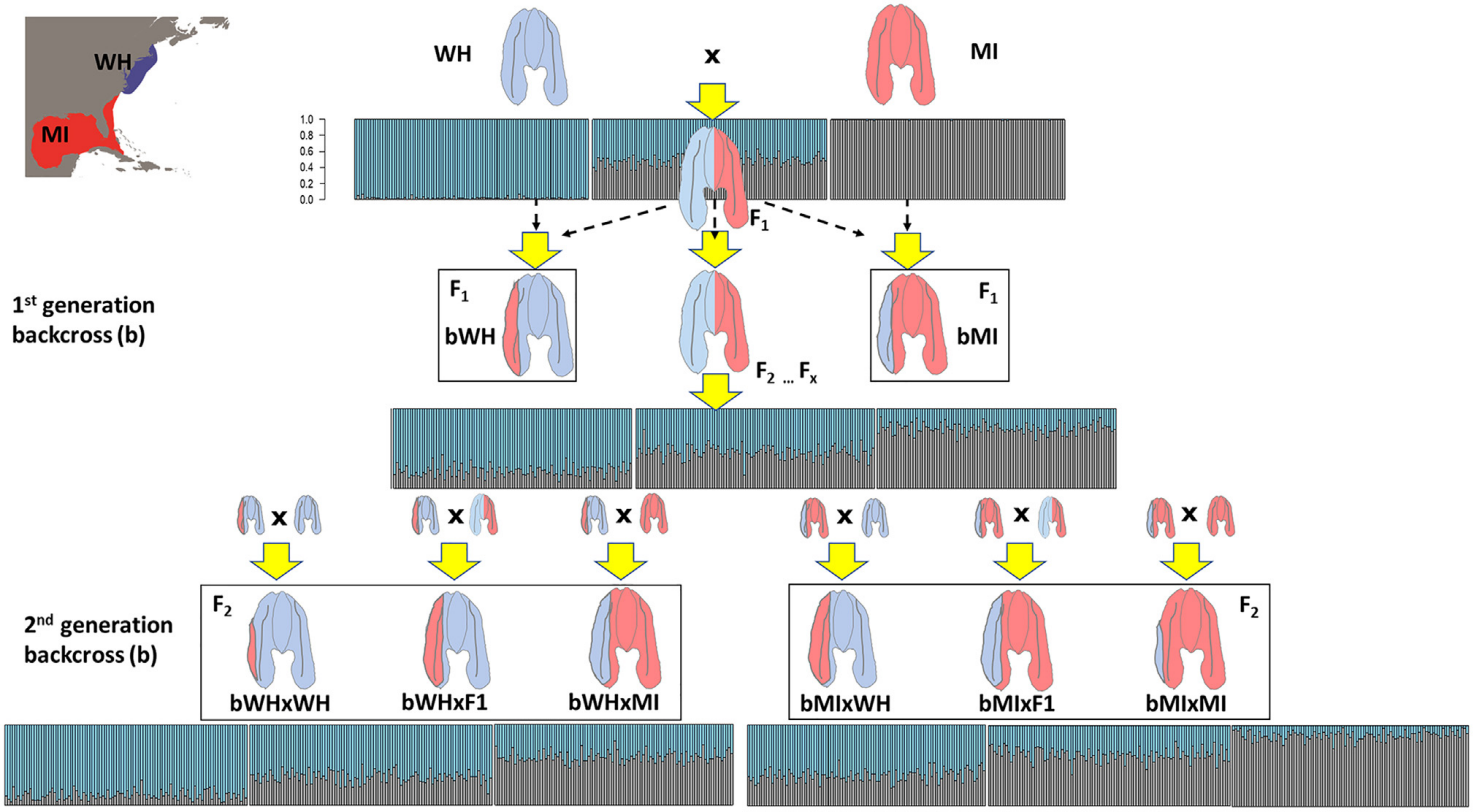
Schematic drawing of F_1_/F_2_ hybrids and 1^st^/2^nd^ generation backcrosses Admixture analysis for all simulated data using STRUCTURE. Individuals were assigned on the basis of the most likely K, in this case (K = 2). A total of 12 categories were simulated: pure northern lineage (WH), pure southern lineage (MI), F_1_ hybrid, F_2_ hybrid, first-generation backcross northern *×* F_1_ (bWH), first-generation backcross southern *×* F_1_ (bMI) and second-generation backcrosses (bWH *×* WH, bWH *×* MI, bWH *×* F_1_, bMI *×* MI, bMI *×* WH and bMI *×* F_1_).

A detailed classification of the hybrids identified in STRUCTURE was conducted in NEWHYBRIDS (Table S2), with potential hybridization scenarios outlined in Figure 5. All specimen collected at locations north of Chesapeake Bay were confirmed as pure northern lineage individuals, while those collected from Miami were confirmed as pure southern lineage individuals.

At the Atlantic coast of Virginia (Wachapreague), samples consisted of 63.6% pure northern individuals, 4.6% F_2_ hybrids, 22.7% first-generation backcrosses, and 9.1% second-generation backcrosses. All first-generation backcrosses were classified as pure northern × F_1_ hybrid backcrosses. Second-generation backcrosses were all classified as backcrosses between first-generation backcrosses (pure northern × F_1_ hybrid) and F_1_ individuals.

In comparison, a higher percentage of admixture was detected inside Chesapeake Bay (Gloucester Point), where only hybrid individuals were identified, i.e., no pure northern or southern individuals. Individuals from Gloucester Point were classified as 30% F_2_ hybrids, 30% first-generation backcrosses (all with pure northern animals) and 40% second-generation backcrosses. Most individuals were classified with high confidence (>90%, see Table S2).

In all comparisons with the locations north of Chesapeake Bay (Sandwich, Woods Hole, Fort Adams, Forth Wetherill, Greenwich Cove, and Esker Point), introgression tests confirmed no significant deviation from the null expectation of D = 0, suggesting no shared ancestry between Miami and the northern locations and no indication of introgression (Table 2). However, we observed an excess shared ancestry between the Atlantic coast station east of Chesapeake Bay (Wachapreague) and Miami, with a c. 2-fold excess of *ABBA* over *BABA* and an average *D* = 0.24 (p < 0.001). This suggests recent introgression from Miami into Wachapreague. Similarly, a highly significant amount of shared ancestry was found between Gloucester Point inside Chesapeake Bay and Miami, with a c. 3-fold excess of *ABBA* over *BABA* and an average *D* = 0.42 (p < 0.001).

**Table 2:** ABBA-BABA tests provide evidence of introgression

P1	P2	P3	ABBA	BABA	*D*	*P*
Sandwich	Woods Hole	Miami	3.04	3.02	0.00	0.98
	Fort Adams	Miami	3.07	2.99	0.01	0.87
	Fort Wetherill	Miami	2.85	1.95	−0.02	0.83
	Greenwich Cove	Miami	3.18	2.96	0.04	0.64
	Esker Point	Miami	2.85	3.02	−0.03	0.70
Woods Hole	Fort Adams	Miami	3.02	2.95	0.01	0.89
	Fort Wetherill	Miami	3.22	2.92	0.05	0.05
	Greenwich Cove	Miami	3.12	2.92	0.03	0.66
	Esker Point	Miami	2.80	2.99	−0.03	0.68
Fort Adams	Fort Wetherill	Miami	3.21	2.97	0.04	0.60
	Greenwich Cove	Miami	3.12	2.98	0.02	0.77
	Esker Point	Miami	2.79	3.04	−0.04	0.54
Fort Wetherill	Greenwich Cove	Miami	3.05	3.15	−0.02	0.84
	Esker Point	Miami	2.74	3.24	−0.08	0.17
Greenwich Cove	Esker Point	Miami	2.71	3.11	−0.07	0.38
Sandwich	Wachapreague	Miami	4.67	2.84	0.24	**<0.001**
Woods Hole		Miami	4.61	2.80	0.25	**<0.001**
Fort Adams		Miami	4.58	2.83	0.24	**<0.001**
Fort Wetherill		Miami	4.49	2.98	0.20	**<0.001**
Greenwich Cove		Miami	4.43	2.83	0.22	**<0.001**
Esker Point		Miami	4.66	2.66	0.27	**<0.001**
Sandwich	Gloucester Point	Miami	6.53	2.64	0.42	**<0.001**
Woods Hole		Miami	6.47	2.59	0.43	**<0.001**
Fort Adams		Miami	6.40	2.59	0.42	**<0.001**
Fort Wetherill		Miami	6.29	2.72	0.40	**<0.001**
Greenwich Cove		Miami	6.27	2.60	0.41	**<0.001**
Esker Point		Miami	6.53	2.46	0.54	**<0.001**

The test is based on the distribution of derived alleles and determines whether introgression has occurred and in which direction. We tested introgression from the southern lineage (Miami) into the northern populations. Significant p values in bold.

## Discussion

The increased resolution of genetic markers sheds new light on the status of hybrids and the nature of introgression between northern and southern lineages of *M. leidyi* in native habitats long the Atlantic coast of the US. We show that hybrids are present at the transition zone between northern and southern lineages, around Cape Hatteras, which represents an oceanographic front where the Gulf Stream begins its arc offshore.^26^ Apart from such oceanographic features that lead to limited gene flow, marine systems are normally characterized by high dispersal and connectivity that usually limits genetic differentiation in marine systems.^31^ *M. leidyi* is an example of a species with high dispersal capacity facilitated by its holoplanktonic life cycle and self-fertilizing reproduction mode.^32^ Hence, high gene flow within areas characterized by high connectivity can be expected, as documented for the invasive range in NW Europe.^23^ In line with this, we observe high gene flow and lack of population structure in the native range north of Chesapeake Bay, which is located north of Cape Hatteras. This is irrespective of diverse habitats, i.e., coastal, estuarine, and offshore waters that were sampled in our study, also including subsequent sampling years. This is concordant with the study of Bayha et al.^25^ using cytochrome *b*, which only detected northern haplotype individuals north of Chesapeake Bay, in an extensive sampling scheme including the Long Island Sound and Rehoboth Bay, DE.

In contrast, two locations just north of Cape Hatteras around/in Chesapeake Bay were highly differentiated from the northern and southern lineages due to the presence of hybrids. In order to understand environmental drivers and document adaptive introgression in hybrid zones, we compare two locations within the hybrid zone with contrasting environments. First, a coastal area along the Atlantic coast characterized by high salinity and relatively stable environmental conditions, and second, Chesapeake Bay, an estuary characterized by high levels of spatial and temporal variability in environmental conditions. The proportion and nature of hybrids found during our investigation were contrasting in the two locations, as outlined in the following sections.

### Stable environmental conditions: Example of a hybrid tension zone

The Atlantic coast east of Chesapeake Bay is characterized by relatively stable environmental conditions, which are more similar to other areas along the north-east coast of the USA.^33^ Salinity in this area, a key environmental parameter, is high (> 30) and relatively constant.^33^ The *M. leidyi* population at this sampling site is composed of pure northern individuals plus a few late-generation hybrids. The population structure found during our investigation is consistent with and suggests a hybrid tension zone. In a hybrid tension zone, the population structure is balanced between dispersal of parental individuals and selection against hybrids. This has been suggested to be due to intrinsic fitness differences of hybrid lines.^12^^,^^16^ Even though we do not have experimental data to proof this hypothesis, our data suggest that hybrids are selected against in this area. On the other hand, lack of hybrids found in more northern sampling locations of New England is probably dispersal-dependent, due to the prevailing current pattern.^26^ Along the north east coast of the US, the coastal current runs toward the south,^26^ which is leading to limited dispersal of animals from the southern lineage to the north, especially considering dispersal beyond Chesapeake Bay. This likely explains why only northern haplotype individuals were detected north of Chesapeake Bay in the most comprehensive previous study using cytochrome *b* as marker gene.^25^

As observed in our data, contact between the northern and southern populations can result not only in hybridization, but also introgression because of backcrossing. However, in a hybrid tension zone, hybrid fitness is expected to be lower relative to the parental forms due to endogenous incompatibilities including the loss of coadapted gene complexes involved in local adaptation due to recombination.^34^ This may explain the low number of hybrids observed in comparison with parental northern genotypes. The lack of southern pure individuals both in pure lines and backcrosses suggests that even though they occasionally arrive from the south, their occurrence is sporadic. Northern lineage animals, in comparison, are expected to be continuously seeded into the Chesapeake Bay area due to the prevailing connectivity pattern with ocean currents running to the south along the coast up to Cape Hatteras.^26^ In support, all backcrosses were from northern individuals. Under the tension zone model, hybrid zones are dynamic and free to move because they are maintained independent of the environment.^35^^,^^36^ This suggests that the stable environment along the Atlantic coast is a hybrid tension zone where selection is likely to act against hybrids due to potentially lower fitness compared to parental lines.

### Variable environmental conditions: Example of a hybrid swarm

A contrasting setting was found inside Chesapeake Bay, where *M. leidyi* is present in a spatially and temporally varying environment, especially when considering salinity, ranging from > 2 to 27.1 PSU (see Figure 4;^30^^,^^37^^,^^42^). Our genetic analyses indicate that no parental forms of either northern or southern origin were detected in Chesapeake Bay. All sampled individuals were of admixed origin, consisting of F_2_ (or later generation) hybrids as well as first- and second-generation backcrosses. This is indicative of a stable hybrid population, actively producing viable offspring and forming a hybrid swarm.^19^^,^^43^ In contrast to the hybrid tension zone model, the prediction of a hybrid swarm is that hybrid fitness is enhanced under particular environmental conditions in which hybrids are superior with increased fitness compared to either of the parental genotypes.^9^^,^^18^^,^^44^ Hybrid superiority can be the result of coadaptation of parental gene pools to distinct exogenous regimes.^8^ Although hybrids might outperform either pure parental species in surviving due to hybrid vigor,^45^^,^^46^ hybrids are not preadapted to the restricted regions where they occur. Their success is rather related to parental forms being less adapted.^8^ In this sense, hybrid swarms are commonly located in marginal habitats substantially different from that of either parental lines.^47^ For instance, natural hybrids between the two North Atlantic eel species are exclusively found in Iceland, characterized by extreme environmental conditions.^21^ In this sense, the hybrid swarm found in our study is in Chesapeake Bay, a large estuary, which is characterized by highly variable environmental conditions, especially considering salinity.^30^ Chesapeake Bay has extensive low salinity (<10) regions where *M. leidyi* is known to form large populations, including in areas of very low salinity (<5).^37^^,^^42^ Salinity has also been linked to differing mortality regimes of *M. leidyi* in Chesapeake Bay; regions of low salinity act as refugia for *M. leidyi* to avoid predation by the scyphomedusa *Chrysaora quinquecirrha*, which is less tolerant to low salinities.^38^ Hence, predation might act as an additional selection pressure to maintain hybrid populations, especially in low saline regions of Chesapeake Bay. Parental forms of the northern linage are not particularly well adapted to low saline conditions, as documented from experimental and field observations in the invasive range.^23^^,^^48^ For the invasive northern population, salinity drastically impacts reproduction rates, and active recruitment in the field ceases at salinities <10.^48^^,^^49^ Salinity effects on reproduction rates in native northern and southern populations have not explicitly been investigated.

One mechanism that might explain the putative fitness advantage of hybrids in the fluctuating environment of Chesapeake Bay is beneficial reversal of dominance, as demonstrated experimentally in the invasive copepod *Eurytemora affinis*.^50^ Dominance reversals are context-dependent so that a beneficial allele in a favorable environment is dominant yet recessive in a non-favored environment.^51^ As the fitness of alleles differs across conditions, beneficial reversals of dominance enable antagonistic selection to maintain high levels of genetic variation for fitness traits such as salinity tolerance,^52^^,^^53^^,^^54^ which would in our case increase the ability to adapt to the fluctuating habitats of Chesapeake Bay.

Hybrid zones are predicted to be ephemeral since their existence is dispersal-dependent.^8^^,^^12^ However, in Chesapeake Bay, hybrids have existed for 20+ years or at least 500 generations. First records date back to 1998, when hybrids were suggested to be present in Rhode River, MD, and then later when sampled in northern and southern Chesapeake Bay areas.^25^ Introgression was observed at all locations, but low marker number did not allow for further hybrid characterization.^25^ Ghabooli et al.^55^^,^^56^ included animals collected in 2008 at York River (same as our study), which appeared highly differentiated in comparison with other native samples, but without testing for hybridization. Moreover, the recent study of Verwimp et al.^40^ using genome wide SNP data, compared genetic variability between native and invasive populations and included a sample location from Chesapeake Bay (N = 7) at the mouth of the Potomac River. Sampling salinity was not provided, but likely ranged around 15. Re-examination of the data by Jaspers et al.^27^ suggested that all individuals from that sampling site were hybrid backcrosses. This indicates that Chesapeake Bay represents a consistent hybrid zone in space and time. Our analyses further suggest that all second-generation backcrosses were between F_1_ hybrids and first-generation backcrosses with pure northern individuals. This indicates that pure northern animals must have been present at low frequencies or originated from nearby coastal areas, with subsequent selection for hybrid lines. Irrespective of the source of the animals observed in Chesapeake Bay, our data suggest the potential presence of a hybrid swarm in this habitat, which is characterized by high environmental variability,^30^ including areas of very low salinity where *M. leidyi* has been found (Figure 4). Even though we cannot exclude that hybrids are intermediate in temperature tolerance, which might contribute to their fitness, salinity is the most extreme environmental parameter in Chesapeake Bay. Taken together, our data suggest that hybrids present in Chesapeake Bay might have a putative adaptive advantage over pure parental lines, but further spatiotemporal population samples and physiological experiments are needed to substantiate this hypothesis.

### Potential role of hybridization in invasiveness

Hybridization in the introduced range as a driver of invasion success has gained considerable attention in invasion ecology. This was originally postulated for plants,^7^ but applies to other organisms as well, including insects,^57^ amphibians,^18^ and fish.^58^ Paradoxically, while recombination in hybrids can generate maladaptive individuals, it can also generate both novel genotypes and an overall increase in genetic variation, which can give hybrids a fitness advantage especially in novel environments.^7^ Moreover, it has been suggested that environmental fluctuations in the native range could facilitate invasion success by imposing balancing selection on key fitness traits.^54^ As a result of balancing selection, native populations from habitats with varying conditions would maintain high standing genetic variation and thereby an enhanced invasive potential.^54^ For example, balancing selection on standing genetic variance for osmotic tolerance in the native range underlies freshwater adaptation in the invasive copepod *E. affinis*.^59^

In the case of *M. leidyi*, hybrids have never been detected in the invasive range either from whole genome^27^ or limited marker set data.^23^^,^^24^^,^^25^^,^^55^^,^^56^^,^^60^ Two scenarios might lead to the presence of *M. leidyi* hybrids in Europe in the future: (1) post-introduction hybridization in the recipient habitat; and (2) translocation of hybrids directly from native habitats to the non-native range. It is interesting to note that the northern and southern *M. leidyi* lineages seem to differ in their salinity tolerance as inferred from their observed distribution range in invasive populations.^23^ While salinity restricts range expansion in Northern Europe,^48^ the southern invasive population thrives at low salinities including the Caspian Sea and the Sea of Azov.^42^ In our study, no pure individuals were genetically identified in Chesapeake Bay. Whether hybrid fitness at low salinity is enhanced over both parental lines needs further investigation via experiments. However, our data suggest that hybrid lines might have an adaptive advantage in low salinity environments and could potentially facilitate range expansion and contribute to the acceleration of their invasion success. We cannot dismiss that other mechanisms such as advection, hydrographic fronts and dispersal differences contribute to maintain hybrid populations in the native range. However, our data suggest that hybrids represent a potential risk if translocated into low saline areas, where *M. leidyi* has not been established, such as the Baltic Sea.^23^ More generally, and unrelated to salinity, hybrid populations can pose a risk due to higher standing genetic variation.^54^ Given the lag time of non-indigenous species in the novel habitat, which has been suggested to facilitate hybridization from distant source pools in the non-native habitat,^7^ translocation from hybrid zones in native areas can be a matter of concern, due to their potential to increase colonization and invasion success in novel habitats. Even though physiological experiments and increased spatiotemporal samplings are needed to confirm the extent of the here detected hybrid zone of *M. leidyi* in the native range, this study contributes to the general understanding of how hybridization in native populations might contribute to successful invasions in the marine environment. We encourage genomic monitoring of native populations of highly invasive species in order to identify hybrid populations to prevent translocation of admixed individuals from hybrid zones in the native area to new, thus far uninvaded habitats.

### Limitations of the study

We are the first to disentangle the status and proportion of admixed individuals and the nature of the hybrid zone in the native habitat of *M. leidyi* using a large number of genetic markers. We acknowledge that our spatial and temporal sampling of Chesapeake Bay is limited. At present, our study does not include the very low saline locations which would be needed to further support a putative advantage of hybrids in Chesapeake Bay, while temporal samples would allow to assess the overall stability of the hybrid zones. However, previous studies using low number of markers^25^^,^^40^^,^^55^^,^^56^ confirm that the hybrid zone in Chesapeake Bay has existed for at least 20 years. Future studies should include extended sampling using genome-wide markers also in area between New England and Chesapeake Bay, where hybridization is unlikely but might occur. As discussed, the observation of hybrids in Chesapeake Bay points to a putative hybrid advantage in the particular variable environmental conditions of Chesapeake Bay. While other factors might contribute to the observed population structure (dispersal, temperature), we hypothesize that salinity in combination with predation pressure by the higher saline adapted jellyfish *Chrysaora quinquecirrha* are likely the drivers to maintain hybrids in Chesapeake Bay. Experiments should be conducted to substantiate a hybrid advantage at extreme salinity levels.

## STAR★Methods

### Key resources table

**Table:** 

REAGENT or RESOURCE	SOURCE	IDENTIFIER
**Biological samples**		

DNA from M. leidyi	This study	N/A
DNEasy Blood & Tissue kit	Qiagen	Cat#69504
Fluidigm 96.96 Dynamic Arrays	Fluidigm Corporation	N/A
Fluidigm SNP Genotyping Analysis software	Fluidigm Corporation	N/A

**Deposited data**		

SNP data	Dryad	https://doi.org/10.5061/dryad.dv41ns23z
R Code	Zenodo	https://doi.org/10.5281/zenodo.8238979

**Software and algorithms**		

Arlequin	Excoffier and Lischer^61^	http://cmpg.unibe.ch/software/arlequin35
SmartPCA	Patterson et al.^62^	https://github.com/chrchang/eigensoft/blob/master/POPGEN
Structure	Pritchard et al.^63^	https://web.stanford.edu/group/pritchardlab/structure.html
Newhybrids	Anderson and Thomas^64^	https://ib.berkeley.edu/labs/slatkin/eriq/software/software.htm#NewHybs

### Resource availability

#### Lead contact

Further information and requests should be directed to and will be fulfilled by the lead contact, Cornelia Jaspers (coja@aqua.dtu.dk).

#### Materials availability

This study did not generate unique reagents.

#### Data and code availability

• All SNP data have been deposited at Dryad and are publicly available at: https://doi.org/10.5061/dryad.dv41ns23z.
• All original code has been deposited at Zenodo and is publicly available at: https://doi.org/10.5281/zenodo.8238979.
• Any additional information required to reanalyze the data in this paper is available from the lead contact upon request.

### Experimental model and subject details

#### Sample collection

All permissions and regulations to sample the invertebrate comb jelly (ctenophore) *Mnemiopsis leidyi* in the native range along the US east coast have been attained and were followed. No cultivation was needed for sample generation. *M. leidyi* is a simultaneous hermaphrodite hence sex bias does not apply. Specifically, a total of 176 *M. leidyi* individuals were collected at eight locations along the US Atlantic coast from Cape Cod to Chesapeake Bay: Sandwich (MA, N = 34), Woods Hole (MA, N = 25), Fort Adams (RI, N = 35), Fort Wetherill (RI, N = 25), Greenwich Cove (RI, N = 7), Esker Point (CT, N = 18), Wachapreague (VA, N = 22) and in Chesapeake Bay at Gloucester Point (VA, N = 10) (see Table S3 for location and environmental details; Figure 1). Salinity environments encountered by *M. leidyi* in the Northern population investigated here (USA-states: MA, RI, CT) are high and >19 even in Narraganset Bay,^65^ while in Chesapeake Bay, *M. leidyi* has been confirmed from salinities as low as >2 (Figure 4).

Samples were collected at all locations during late summer/early fall 2018 (N = 102), additional samples were collected at four locations in summer/early fall 2020 (N = 74) to allow for temporal genetic analysis. All samples were collected north of Cape Hatteras and correspond to the northern lineage identified in previous genetic studies.^25^^,^^27^ For comparison, we also included samples collected at one site south of Cape Hatteras (Miami, FL, N = 15), which corresponds to the southern lineage previously analyzed in Jaspers et al.^27^ The latter is the only southern native population analysed using whole genome sequencing data up to now. Previous analyses using low genetic marker density showed differences between Gulf of Mexico and Florida *M. leidyi* populations for mitochondrial cytochrome b, but not for microsatellite markers.^25^ However, connectivity between the locations previously analysed along the US Gulf of Mexico coastline^25^ and Florida, Miami is limited. We can not exclude that cryptic diversity exists in the southern linage but so far, no differentiation has been found within the southern Atlantic linage from south of Cape Hatteras to Florida.^25^

### Method details

#### DNA extraction

*M. leidyi* were individually placed on coffee filters and dried for 48 hours at 60°C. DNA was extracted using the DNEasy Blood & Tissue kit (Qiagen) following the manufacturer’s protocol except for the sample processing and the elution steps. A 1 cm x 1 cm piece of dried tissue was cut from coffee filters and placed into a 2 ml microcentrifuge tube. 180 μl ATL buffer and 20 μl proteinase K were added, mixed by vortexing and incubated for 3 hours at 56°C with occasional vortexing in between. After centrifuging at 10,000 rpm for 1 min, 200 μl AL buffer was added, mixed thoroughly by vortexing and incubated at room temperature for 10 min. In the final elution step, after discarding the collection tube and transferring the column into a new 1.5 ml Eppendorf tube, DNA was eluted by adding 50 μl AE buffer (pre-heated to 60°C) to the center of the spin column membrane, incubating for 2 min and centrifuging at 10,000 rpm for 1 min. DNA concentration and purity were measured and afterwards samples were diluted 1:100 for further analyses.

### Quantification and statistical analysis

#### SNP chip genotyping

All individuals were genotyped at a total of 96 single nucleotide polymorphisms (SNPs) using a high throughput low-density SNP microarray, developed from whole-genome re-sequencing data.^29^ SNP genotyping was performed using Fluidigm 96.96 Dynamic Arrays (Fluidigm Corporation, San Francisco, CA, USA). The Fluidigm system uses nano-fluidic circuitry to allow for the simultaneous genotyping of up to 96 samples with 96 loci.^66^ Genotypes were called and compiled using the Fluidigm SNP Genotyping Analysis software. Each assay was assessed for plot quality and expected clustering patterns. Northern and southern lineage individuals identified in Jaspers et al.^27^ were used as positive controls.

#### Data analyses

Genetic diversity was estimated using observed (Ho) and expected (He) heterozygosities and standardized allelic richness (AR) per population, calculated in Arlequin v3.5.2.2.^61^ Diversity values across populations were compared by one-way ANOVA using R. Standardized genetic differentiation statistics between sampling locations were calculated using Arlequin v3.5.2.2 in accordance with Weir and Cockerham.^67^ First, pairwise genetic differentiation (F_ST_) was calculated between all sample pairs. Second, a hierarchical AMOVA was conducted partitioning genetic differentiation into a geographical and temporal component.

All SNP data were used to conduct a Principal Component Analysis (PCA) to visualize population structure using smartPCA from the Eigensoft package,^62^ with significance calculated using the Tracy-Widom statistic.^68^ Population structure was further investigated using the Bayesian assignment approach implemented in STRUCTURE,^63^ a model-based clustering algorithm that infers the most likely number of groups (K) in the data. The analysis was performed with K = 1–9, assuming an admixture model, correlated allele frequencies and without population priors. A burn-in of 100,000 steps followed by 1,000,000 additional Markov Chain Monte Carlo iterations were performed. For each K, 10 independent runs were conducted to check the consistency of the results. The most likely K was inferred using the method of Evanno et al.,^69^ which measures the steepest increase of the ad hoc statistic ΔK based on the rate of change in the log probability of data between successive K values. STRUCTURE was also used to identify hybrids, estimating individual admixture proportions and their probability intervals.

Hybridization patterns were assessed using the framework of Bayesian model-based clustering implemented in NEWHYBRIDS,^64^ which computes the posterior probability of belonging to each of the parental and distinct hybrid classes. The original genotype classes: parental northern, parental southern, F1 (parental northern x parental southern), F2 (F1 x F1) and first-generation backcrosses (F1 x parental northern, F1 x parental southern) were expanded to include all possible second-generation backcrosses (see Figure 5). The software was run for 100,000 iterations in the burn-in period, followed by one million Markov Chain Monte Carlo iterations in each analysis.

Introgression was also investigated by testing for an excess of shared derived alleles using the ABBA-BABA test.^70^ The test considers ancestral (A) and derived (B) alleles and given three populations (P1, P2, P3) and an outgroup O with the relationship (((P1,P2),P3),O), counts the SNPs that match the ABBA and BABA genotype patterns. An excess of ABBA is indicative of recent introgression from P3 into P2, while an excess of BABA suggests excess shared ancestry between P1 and P3. Excess of ABBA or BABA patterns was tested by calculating Patterson’s *D* statistic^71^ using a jackknife method to test for a significant deviation from the null expectation of *D* = 0. In our case, we used the baseline population of Miami as P3 in order to infer the amount of shared ancestry between the northern populations and Miami and the direction of introgression. We included the North Sea population from Jaspers et al.^27^ as an outgroup.

#### Power of the markers to identify hybrids

To test the power of the markers to classify hybrids, a total of 120,000 individuals were simulated using all SNPs in the dataset for 12 categories including first- and second-generation backcrosses and reassigned blindly. Figure 5 shows all STRUCTURE plots for all simulated categories. Using NEWHYBRIDS, a correct assignment was made for all parental individuals, both northern and southern lineages, with 100% confidence (Table S4). Identification of F_1_ vs. F_2_ hybrids was more difficult since both classes shared similar admixture proportions and F_1_ hybrids presented no exclusive genotypes relative to F_2_ hybrids. However, F_2_ hybrids could be distinguished from F_1_ hybrids by the presence of recombinant genotypes and correctly assigned with a confidence of 94%. Regarding backcrosses, a correct assignment with high confidence was obtained for first-generation (on average 90.4%) and second-generation backcrosses with the same parental type (on average 94.4%). The remaining second-generation backcrosses were correctly assigned with lower confidence (60.1-61.8%). Overall, results suggest that our SNP panel has enough discriminatory power to correctly identify parental northern, parental southern, F_2_, first-generation and second-generation hybrids. While simulated F_1_ hybrids could also be assigned as F_2_ hybrids, they could not be assigned as parental or backcrosses. It should also be noted that we extended the original hybrid classes to include second-generation backcrosses but we did not include later generation hybrids such as F_3_ or F_4_ hybrids, which would be not possible to distinguish from F_2_ hybrids, hence we refer to those hybrids as F_2_ (or later generation) hybrids in the discussion.
