# Supplementary material for: Hybridization and adaptive introgression in a marine invasive species in native habitats
Source: iScience. 2023 Nov 10;26(12):108430. doi: 10.1016/j.isci.2023.108430 (PMC10698267; doi:10.1016/j.isci.2023.108430)
Supplement: Document S1. Figure S1 and Tables S1–S4 [file mmc1.pdf]

iScience, Volume *26*

Supplemental information

Hybridization and adaptive introgression

in a marine invasive species in native habitats

José Martin Pujolar, Denise Breitburg, Joanna Lee, Mary Beth Decker, and Cornelia Jaspers

Supporting online information:

Figure S1. Admixture analysis visualized by STRUCTURE plots using alternative clusters with K = 3 to 5. Related to Figure 3.

Abbreviations: SA = Sandwich, MA; WH = Woods Hole, MA; FA = Fort Adams, RI; FW = Fort Wetherill, RI; GC = Greenwich Cove, RI; EP = Esker Point, CT; WA= Wachapreague, VA; GP = Gloucester Point, Chesapeake Bay, VA; MI = Miami, FL.

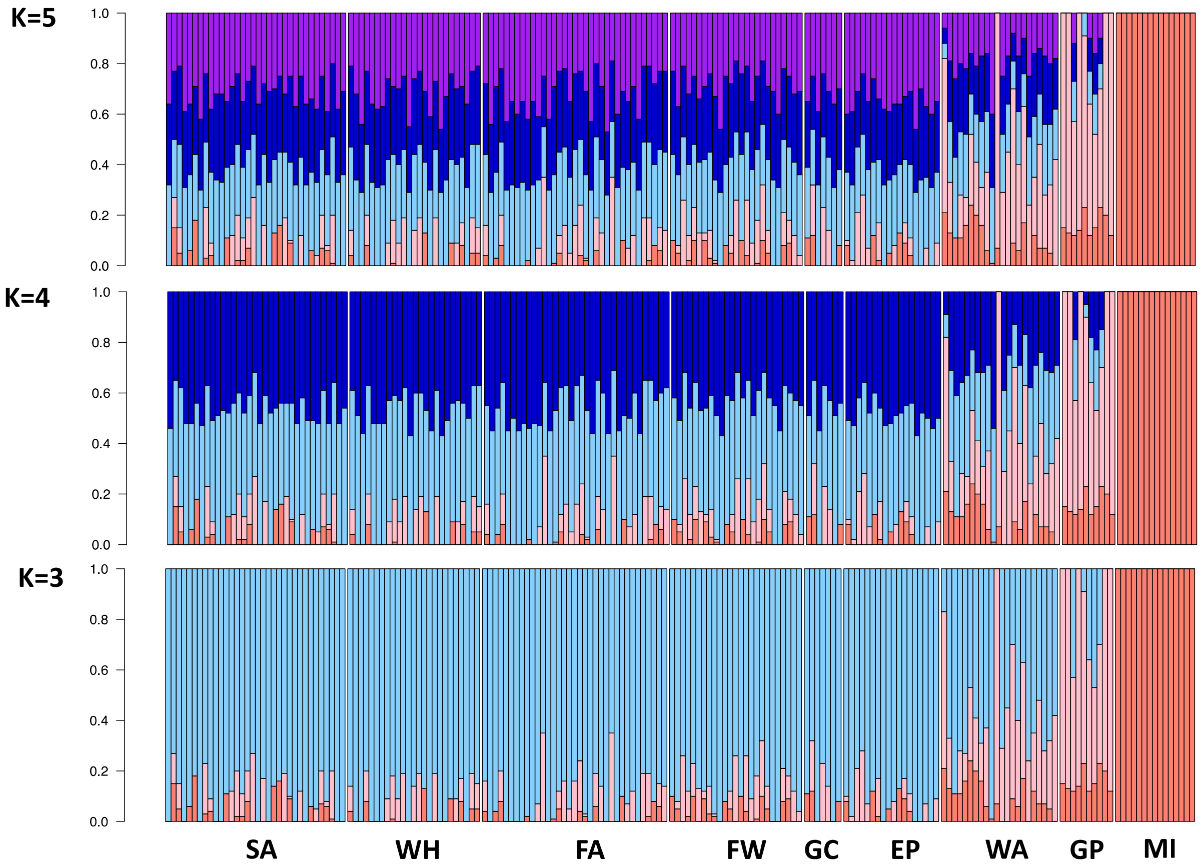

Table S1. Summary of genetic diversity. Related to Figure 1.

Indices of genetic diversity across sampling locations, including observed (H_o) and expected (H_e) heterozygosities and allelic richness (AR). N=number of individuals. SD=standard deviation.

Location	N	H_o	SD	H_e	SD	AR	SD
**Sandwich, MA**	34	0.288	0.169	0.295	0.145	1.913	0.283
**Woods Hole, MA**	25	0.307	0.175	0.314	0.128	1.870	0.339
**Fort Adams, RI**	35	0.322	0.183	0.313	0.143	1.891	0.313
**Fort Wetherill, RI**	25	0.320	0.174	0.313	0.143	1.880	0.326
**Greenwich Cove, RI**	7	0.347	0.193	0.338	0.143	1.739	0.442
**Esker Point, CT**	18	0.270	0.162	0.311	0.141	1.837	0.371
**Wachapreague, VA**	22	0.338	0.174	0.338	0.138	1.902	0.299
**Gloucester Point, VA**	10	0.359	0.212	0.353	0.138	1.848	0.361
		P =0.921		P =0.948		P =0.696	

Table S2. Assignment probability in NEWHYBRIDS of all individuals from Wachapreague and Gloucester Point. Related to Figure 1. Categories included: pure northern lineage (WH), pure southern lineage (MI), F_1 hybrid, F_2 hybrid, first-generation backcross northern×F_1 (bWH), first-generation backcross southern×F_1 (bMI) and second-generation backcrosses (bW×WH, bWH×MI, bWH×F_1, bMI×MI, bMI×WH and bMI×F_1).

Region	Nr.	Class	*P*	Alt. class I	*P*	Alt. class II	*P*
**Atlantic coast**	1	F_2	0.87	bWHxF_1	0.10	F_1	0.02
(Wachapreague)	2	WH	1.00				
	3	WH	1.00				
	4	WH	1.00				
	5	WH	0.78	bWHxWH	0.22		
	6	bWHxF_1	0.90	F_2	0.07	bMIxWH	0.03
	7	bWH	1.00				
	8	bWH	1.00				
	9	WH	0.85	bWHxWH	0.15		
	10	WH	1.00				
	11	bWHxF_1	0.85	F_2	0.10	bMIxWH	0.05
	12	WH	1.00				
	13	WH	1.00				
	14	bWH	1.00				
	15	WH	1.00				
	16	bWH	1.00				
	17	WH	1.00				
	18	WH	0.87	bWHxWH	0.13		
	19	bWH	0.96	bWHxWH	0.04		
	20	WH	1.00				
	21	WH	1.00				
	22	WH	1.00				
**Chesapeake Bay**	1	F_2	0.70	F_1	0.30		
(Gloucester Point)	2	F_2	0.73	F_1	0.27		
	3	bWH	1.00				
	4	F_2	0.78	F_1	0.21	bMIxWH	0.01
	5	bWHxF_1	0.90	F_2	0.07	bMIxWH	0.03
	6	bWH	1.00				
	7	bWH	1.00				
	8	bWHxF_1	0.98	bMIxWH	0.02		
	9	bWHxF_1	0.96	bMIxWH	0.04		
	10	bWHxF_1	0.98	bMIxWH	0.02		

Legend:

Pure line north	WH
Pure line south	MI
Hybrid 1st generation	F_1
Hybr. 2nd & higher gen.	F_2-F_x
1st gen. backcross (b)	bWH
2nd gen. backcross	bWHxF_1

Table S3. Sampling details of the study. Related to Figure 1.

Details of genetic samples of *M. leidyi* including sampling location, geographical coordinates, salinity, sampling date and number of individuals collected per location.

State	Location	Latitude	Longitude	Salinity	Date	N
Massachusetts	Sandwich	41.77	-70.48	35	21/09/2018	11
					14/09/2020	19
					19/10/2020	4
	Woods Hole	41.53	-70.68	33	07/09/2018	7
					14/09/2018	8
					10/09/2020	10
Rhode Island	Fort Adams	41.47	-71.34	32	28/08/2018	6
					21/10/2018	8
					29/09/2020	14
					03/10/2020	7
	Fort Wetherill	41.48	-71.36	34	11/09/2018	5
					08/09/2020	8
					08/10/2020	12
	Greenwich Cove	41.65	-71.45	30	19/09/2018	7
Connecticut	Esker Point, Groton	41.32	-72	28-31	30/10/2018	18
Virginia	Wachapreague, Atlantic coast east of Chesapeake Bay	37.6	-75.66	30.3	25/07/2018	22
	Gloucester Point Beach Park, inside Chesapeake Bay	37.25	-76.5	18	23/08/2018	10

Table S4. Assignment probability of simulated individuals in NEWHYBRIDS. Related to Figure 5.

A total of 12 categories were simulated: pure northern lineage (WH), pure southern lineage (MI), F_1 hybrid, F_2 hybrid, first-generation backcross northern× F_1 (bWH), first-generation backcross southern×F_1 (bMI) and second-generation backcrosses (bWH×WH, bWH×MI, bWH×F_1, bMI×MI, bMI×WH and bMI×F_1).

	**WH**	**bWHxWH**	**bWH**	**bWHxF_1**	**bMIxWH**	**F_2**	**F_1**	**bMIxF1**	**bWHxMI**	**bMI**	**bMixMI**	**MI**
**WH**	**0.956**	0.040	-	-	-	-	-	-	-	-	-	-
**bWHxWH**	0.044	**0.922**	0.065	-	-	-	-	-	-	-	-	-
**bWH**	-	0.038	**0.905**	0.044	-	-	-	-	-	-	-	-
**bWHxF_1**	-	-	0.030	**0.618**	0.391	0.033	-	-	-	-	-	-
**bMIxWH**	-	-	-	0.338	**0.609**	-	-	-	-	-	-	-
**F_2**	-	-	-	-	-	**0.940**	**0.500**	-	-	-	-	-
**F_1**	-	-	-	-	-	-	**0.500**	-	-	-	-	-
**bMIxF_1**	-	-	-	-	-	0.037	-	**0.603**	0.038	0.051	-	-
**bWHxMI**	-	-	-	-	-	-	-	0.373	**0.601**	0.033	-	-
**bMI**	-	-	-	-	-	-	-	0.034	0.021	**0.903**	0.438	-
**bMixMI**	-	-	-	-	-	-	-	-	-	0.024	**0.962**	-
**MI**	-	-	-	-	-	-	-	-	-	-	-	**1.000**
